# Supplementary material for: Systematic Review of the Empirical Evidence of Study Publication Bias and Outcome Reporting Bias
Source: PLoS One. 2008 Aug 28;3(8):e3081. doi: 10.1371/journal.pone.0003081 (PMC2518111; doi:10.1371/journal.pone.0003081)
Supplement: Appendix S1 — Search Strategy. (0.03 MB DOC) [file pone.0003081.s001.doc]

Appendix 1 Search Strategy

Medline search strategy (1950 to December 2007)

1. publication$.tw.

2. exp Publications/

3. publish$.tw.

4. exp Publishing/

5. 1 or 2 or 3 or 4

6. bias.tw.

7. exp "Bias (Epidemiology)"/

8. 6 or 7

9. 5 and 8

10. exp Publication Bias/

11. 9 or 10

12. selective report$.tw.

13. selective non report$.tw.

14. selective non-report$.tw.

15. outcome report$ bias.tw.

16. 12 or 13 or 14 or 15

17. 11 or 16

18. cohort.tw.

19. exp Cohort Studies/

20. randomized controlled trial$.tw.

21. randomised controlled trial$.tw.

22. 18 or 19 or 20 or 21

23. 17 and 22

**Scopus search strategy** (1960 to December 2007)

1. TITLE-ABS-KEY(publications$)
2. TITLE-ABS-KEY(publish$)
3. (TITLE-ABS-KEY(publications$)) OR (TITLE-ABS-KEY(publish$))
4. TITLE-ABS-KEY(bias)
5. ((TITLE-ABS-KEY(publications$)) OR (TITLE-ABS-KEY(publish$))) AND (TITLE-ABS-KEY(bias))
6. TITLE-ABS-KEY(selective report$)
7. TITLE-ABS-KEY(selective non report$)
8. TITLE-ABS-KEY(selective non-report$)
9. TITLE-ABS-KEY(outcome report$ bias)
10. (TITLE-ABS-KEY(selective report$)) OR (TITLE-ABS-KEY(selective non report$)) OR (TITLE-ABS-KEY(selective non-report$)) OR (TITLE-ABS-KEY(outcome report$ bias))
11. (((TITLE-ABS-KEY(publications$)) OR (TITLE-ABS-KEY(publish$))) AND (TITLE-ABS-KEY(bias))) OR ((TITLE-ABS-KEY(selective report$)) OR (TITLE-ABS-KEY(selective non report$)) OR (TITLE-ABS-KEY(selective non-report$)) OR (TITLE-ABS-KEY(outcome report$ bias)))
12. TITLE-ABS-KEY(cohort)
13. TITLE-ABS-KEY(randomized controlled trial$)
14. TITLE-ABS-KEY(randomised controlled trial$)
15. (TITLE-ABS-KEY(cohort)) OR (TITLE-ABS-KEY(randomized controlled trial$)) OR (TITLE-ABS-KEY(randomised controlled trial$))
16. ((((TITLE-ABS-KEY(publications$)) OR (TITLE-ABS-KEY(publish$))) AND (TITLE-ABS-KEY(bias))) OR ((TITLE-ABS-KEY(selective report$)) OR (TITLE-ABS-KEY(selective non report$)) OR (TITLE-ABS-KEY(selective non-report$)) OR (TITLE-ABS-KEY(outcome report$ bias)))) AND ((TITLE-ABS-KEY(cohort)) OR (TITLE-ABS-KEY(randomized controlled trial$)) OR (TITLE-ABS-KEY(randomised controlled trial$)))

The Cochrane Methodology Register Search strategy (1898 to December 2007)

1. [(publication bias):kw or (outcome reporting bias):kw in Methods Studies](http://www3.interscience.wiley.com/cochrane/searchHistory?mode=runquery&qnum=5)
