# Supplementary material for: Systematic Review of the Empirical Evidence of Study Publication Bias and Outcome Reporting Bias
Source: PLoS One. 2008 Aug 28;3(8):e3081. doi: 10.1371/journal.pone.0003081 (PMC2518111; doi:10.1371/journal.pone.0003081)
Supplement: Text S1 — Explanation of flow diagram. (0.03 MB DOC) [file pone.0003081.s002.doc]

**Text box 1: Explanation of flow diagram**

Flow diagrams were constructed to describe the process of a study from approval through to publication, including the publication of outcomes. There are several routes a study can follow. Once approved, a study is either carried out and completed, stopped early or abandoned after it has been approved. A study may have stopped early due to patient accrual or reasons to do with the trial investigator or funding problems. Randomised controlled trials may be stopped early due to results from interim analyses, so these along with completed studies (plus in some cases other studies that are stopped early or ongoing studies) carry on to the next stage – submission to a journal.

If a report of a study is submitted to a journal, it can then either be accepted or rejected. However, acceptance leads to full publication in a journal whereas rejection again leads to non-publication (taking into account rejection from all journals submitted to). Some studies are presented only in abstract form, for example at a conference; these are in the public domain and could be found if a thorough search were to be carried out. Although abstracts may be difficult to locate and we can have no expectation of full reporting of outcomes and analyses, they are helpful to identify that a trial was conducted and contact with the trialists for further information would be necessary.

When a study is published, there can be several types of selective reporting of outcomes [10]. We have concentrated on the primary outcome of the trial and whether it is fully reported, partially reported (e.g. only as a p-value) or not reported. Outcomes specified in the protocol can be compared to those in the publication with regard to their status as primary or secondary, whether they have been omitted or new outcomes added, or whether they are partially reported. However, some studies may have several primary outcomes and some studies may have no specified primary outcome. In such circumstances, how we tackle this will depend on how each researcher handled data in their own cohort.
